# Dystonia: opportunities to gain insights into underlying pathophysiological mechanisms

**DOI:** 10.1007/s00415-017-8411-5

**Published:** 2017-02-10

**Authors:** K. J. Peall, N. P. Robertson

**Affiliations:** Institute of Psychological Medicine and Clinical, Neurosciences, Cardiff University, Cardiff, CF14 4XW UK

## Introduction

Dystonia is one of the most common movement disorders, a core component of the isolated and combined dystonias as well as contributing to the motor phenotype of several neurodegenerative movement disorders, such as Parkinson’s disease and Huntington’s disease. In spite of this, the pathophysiological mechanisms underlying dystonia remain poorly understood with the current thinking centered on a circuit-based disorder, with altered synaptic plasticity impacting higher neuronal circuits and networks. In this review, we discuss three publications with distinct approaches in attempting to elucidate these mechanisms. These articles also highlight how the combination of gene discovery, cellular assays, and systems-based analysis can each contribute to building an understanding of complex disorders.

## Mutations in the histone methyltransferase gene *KMT2B* cause complex early onset dystonia

This paper describes a stepwise approach to a novel gene discovery and confirmation of pathogenesis in a cohort of patients with an undiagnosed childhood-onset dystonia. Ten individuals were identified with overlapping heterozygous interstitial microdeletions involving a region on chromosome 19 containing two genes, *KMT2B* and *ZBTB32*. The next generation sequencing techniques identified *KMT2B* heterozygous variants in six further cases, a single case by Sanger sequencing, and a further ten cases from national and international collaborations.

Meyer and colleagues provide a detailed description of the clinical characteristics and investigational findings of those identified with *KMT2B* mutations. The majority had an early childhood onset (1–9 years) of dystonia symptoms with no evidence of gender bias. The dystonic symptoms began predominantly in the craniocervical region or limbs, progressing to a more generalized form 2–11 years after presentation. Additional clinical features included characteristic facial appearances (elongated face and bulbous nasal tip), developmental delay, intellectual disability, and systemic symptoms involving skin, renal, and respiratory symptoms. Cerebral imaging identified symmetrical hypointensity of the globus pallidi, although no abnormalities were observed following neurotransmitter analysis of CSF. Deep brain stimulation (DBS) in ten cases provided symptomatic improvement, whilst oral medical therapy was ineffective. No overt genotype–phenotype relationship was immediately observed, although those with the rarer missense mutations tended to develop symptoms at a later age.

A key component of a novel gene discovery is demonstrating that the mutations are likely to impact the normal function of the encoded protein. *KMT2B* encodes a histone lysine methyltransferase that is involved in the methylation of histone 3 at lysine 4 (H3K4), an important epigenetic modification associated with active gene transcription. In silico homology modeling techniques predicted that a number of the identified variants would impact the structure–function properties of the KMT2B protein. Analysis of healthy donor adult and foetal tissue found the protein to be ubiquitously expressed throughout the brain, with the highest levels observed in the cerebellum, while quantitative RT-PCR of patient-derived fibroblasts demonstrated overall lower KMT2B levels than controls. Fibroblast and CSF protein expression analysis were also used to determine whether *KMT2B* mutations could impact on other known dystonia-related pathways. These identified reduced *THAP1* and *TorsinA* transcripts (genes involved in DYT6 and DYT1 dystonia, respectively), a reduction in CSF dopamine-2-receptor (D2R) and increased Tyrosine Hydroxylase levels.

*Comment: KMT2B* has been given the DYT28 identifier, making it the most recent in a growing number of disease-causing genes for dystonia. The ongoing development and reducing costs of the next generation sequencing techniques suggest that this number is likely to continue to rise, providing increasing opportunity to understand the mechanisms that underpin this complex set of disorders. Although mutations in each of these individual genes remain rare, their identification and subsequent interrogation of function on a cellular and system-based level provide an opportunity for the identification of overlapping pathways, or points of commonality that may be amenable to therapeutic intervention.

Meyer E et al (2017) Nat Genet 49(2):223–237.

## Functional genomic analyses of mendelian and sporadic disease identify impaired elF2α signaling as a generalizable mechanism for dystonia

This paper outlines the stepwise approach taken to identifying, and building upon, the evidence for elF2α signaling pathway involvement in Mendelian and seemingly sporadic forms of dystonia.

An initial step involved a successful validation of a cellular-based assay for DYT1 dystonia (GAG deletion in *TorsinA*) based on a known Torsin1a biology. Expression of wild type and mutant Torsin1a in human embryonic kidney (HEK) cells demonstrated a distinct and readily identifiable pattern of protein expression. Having established a reliable cellular assay, small interfering RNAs (siRNAs) were utilized to undertake a hypothesis-free genome-wide screen. siRNAs are double-stranded RNA molecules that interfere with gene expression by degrading post-transcriptional mRNA and prevent translation to the protein product. Using a quality control (QC) index of a minimum three standard deviation improvement to Torsin1a localization, whole genome screen and subsequent pathway analysis identified 93 high stringency and high-quality genetic hits. Further validation identified 11 overrepresented signaling pathways with the elF2α signaling pathway the most enriched.

The elF2α pathway is a ubiquitous cellular pathway known as the integrated stress response (ISR). ElF2α phosphorylation is thought to contribute to synaptic plasticity, being required for long-term depression (LTD) at cortico-striatal synapses. The rate-limiting elF2α complex subunit is phosphorylated by one of four upstream stress-sensitive kinases, knock down of which resulted in increased abnormal Torsin1a localization, indicating that it is decreased activity of the signaling pathway that contributes to pathogenesis. Pharmacological testing provided further confirmation still with elF2α phosphorylation inhibitors (e.g., Salubrinal) normalizing Tosrin1a localization in a dose-dependent manner. Compounds targeting other signaling pathways identified in the whole genome screen (notch and glucocorticoid signaling) found no effect, suggesting that these effects were specific to the elF2α pathway.

ElF2α phosphorylation also appears to be involved in the function of other genes implicated in the pathogenesis of dystonia. Whole exome sequencing of 20 unrelated individuals with sporadic isolated dystonia identified 12 exons with rare coding variants, one of which being the first exon of *ATF4*, one of several genes upregulated by elF2α phosphorylation. Sanger sequencing of 239 sporadic cervical dystonia cases found *ATF4* functional variants in 8.8% (3/239) cases compared to 0.5% in controls. Within the DYT1 sample, patient-derived fibroblasts also demonstrated reduced ATF4 upregulation with basal increases in negative feedback proteins (CReP and GADD34) attenuating the acute phase elF2α signaling. Interestingly, *PRKRA* (mutations of which are seen in DYT16 dystonia) encodes an upstream elF2α kinase activator, with the most common *PRKRA* mutations thought to reduce elF2α phosphorylation.

*Comment:* Overall, this extensive series of experiments provides some preliminary evidence of a common mechanistic pathway, disrupted in distinct ways to give rise to multiple forms of dystonia. This provides a platform to determine not only whether disrupted elF2α signaling is central to other forms of dystonia, but also whether there is a selective brain and/or regional vulnerability to these processes. Moreover, this work provides an exciting inroad for therapeutic development, potentially necessitating treatments that are able to target multiple points within the elF2α pathway.

Rittiner JE et al (2016) Neuron 92:1238–1251.

## Cervical dystonia: a neural integrator disorder

In this publication, Shaikh et al. seek to re-explore the role of the midbrain in the control of head movements, and more specifically cervical dystonia and dystonic tremor. The idea that the midbrain may play a role in the three-dimensional orientation of the head was first suggested in the 1950s, but has gained further traction over the past decade following work addressing the direction and velocity of head movements in individuals with cervical dystonia. This has led to a body of work suggesting a role for a ‘head neural integrator’ located within the midbrain.

The authors first set out their theory from over a decade ago, providing several analogies to ocular motor integrators involved in the control of eye movements, and disruption to which leads to gaze-evoked nystagmus. They also detail the importance of neuronal integration in the medulla and midbrain, more specifically the interstitial nucleus of cajal (INC), in regulating horizontal, vertical, and torsional eye movements. A clear description of the individual components of disrupted eye movements is outlined, with corrective saccades/quick phases being seen when the eyes look in one direction, drifting back involuntarily, and then quickly corrected back to the original direction of gaze. In contrast, the slow-phase velocity changes are dependent on the position of the eye in the orbit, with changes decreasing as the eye position gets closer to the central/straight ahead position, and increasing as they move further from this point.

Klier and colleagues were the first to suggest that an analogous system might be involved in dystonia following pharmacological inactivation of the INC in non-human primates. However, this hypothesis struggled to gain traction at the beginning of the century owing to the lack of involvement of the basal ganglia and/or cerebellum, two regions traditionally considered pivotal in the generation and propagation of dystonia. However, this publication seeks to address some of these conceptual difficulties, extrapolating this original hypothesis to include feedback mechanisms involving the visual system, neck proprioception, and cerebellum to the head neural integrator. Previous work from the same group has shown that vibration of the paraspinal neck muscles increases centripetal head drift during eccentric loading, while others have shown that removal of visual feedback causes the head to drift from the eccentric position towards a central null. The combination of retained visual feedback with vibration-induced distorted proprioception caused the head to drift with rapid corrective movements, resembling dystonic tremor or ‘head nystagmus’.

*Comment:* In spite of the theme of the article, the authors clearly state that these research findings and proposed mechanisms do not necessarily imply that the pathophysiology underlying cervical dystonia is intrinsic to these midbrain regions. Neurophysiological and imaging studies of this region also support an integrative, rather than generator, role for the midbrain with abnormalities in neural excitatory outflow and asymmetry of white-matter projections between the pallidum and the midbrain. Although potentially generating some controversy in challenging the more traditional models of dystonia, this work invites a re-thinking or adjusted view of how dystonia is generated and that similar end phenotypes may be caused by heterogenous underlying biological disruption converging on a common anatomical pathway. Arguably this hypothesis has an additional clinical importance, indicating the need to explore alternative or additional targets for stimulation with DBS and future targeted therapies.

Shaikh AG et al (2016) Brain 139:2590–2599.

